# Levels of adherence to treatment, illness perception and acceptance of illness in patients with coronary artery disease - descriptive and correlational study

**DOI:** 10.1186/s12872-024-03827-w

**Published:** 2024-03-20

**Authors:** Farzad Dugunchi, Shiv Kumar Mudgal, Zohreh Hosseini Marznaki, Hoda Shirafkan, Saeed Abrotan, Fateme Jafarian, Roghayeh Pourkia

**Affiliations:** 1 Student Research Committee, Babol University of Medical Sciences, Babol, Iran; 2 College of Nursing, All India Institute of Medical Sciences, Deoghar, India; 3 Imam Ali Hospital, Amol, Mazandaran University of Medical Sciences, Sari, Islamic Republic of Iran; 4 Social Determinants of Health Research Center, Health Research Institute, Babol University of Medical Sciences, Babol, Iran; 5 Department of Cardiology, Clinical Research Development Unit of Rouhani Hospital, Babol University of Medical Sciences, Babol, Iran; 6 Department of Cardiology, School of Medicine, Babol University of Medical Sciences, Babol, Iran

**Keywords:** Coronary artery disease, Acceptance of illness, Medication adherence, Illness perception

## Abstract

**Background:**

Understanding the disease and its acceptance significantly influence adherence to prescribed medications, a critical aspect in managing coronary artery disease (CAD). This study is designed to explore the multifaceted factors influencing medication adherence specifically in CAD patients. Of particular interest is investigating the interconnectedness between medication adherence, the perception of illness, and the level of acceptance of the illness itself among these individuals.

**Methods:**

This cross-sectional study involved 280 confirmed CAD patients who were selected through a convenience sampling method adhering to predefined inclusion criteria. The study was conducted between March and September 2023. Three primary parameters—medication adherence, illness perception, and acceptance of illness—were evaluated using standardized tools: The Morisky Medication Adherence Scale-8, Illness Perception Questionnaire-Brief, and Acceptance of Illness Scale. Statistical analyses using SPSS (version 25) were used to analyze the data.

**Results:**

Patients had moderate illness perception (51.82 ± 7.58) and low acceptance to illness (16.98 ± 4.75), and 61.8 of them adhered to their medication regimen. A positive relationship between acceptance of illness and medication adherence (*r* = 0.435, *p*-value < 0.01) was found. Level of education, type of drug and marital status had significantly impact on medication adherence, and gender, level of education, intention to stop drug and marital status were associated with acceptance of illness (*p* < 0.05).

**Conclusion:**

These results underscore the pivotal role of medication adherence in CAD management. Future interventions should target improving illness perception and acceptance of illness among CAD patients to enhance their overall adherence to prescribed medications and ultimately improve disease management.

## Introduction

Coronary artery disease (CAD) remains a significant contributor to global mortality, ranking as the third leading cause of death worldwide and accounting for an estimated 17.8 million annual fatalities [1]. Specifically in Iran, CAD holds the foremost position as the primary cause of death, attributing to approximately 50% of the annual mortality rate [2]. This widespread affliction places substantial societal burdens in terms of economic costs, disease burden, and escalated mortality rates [3]. Given the chronic and progressive nature of CAD, effective disease control and management become paramount [4]. Medication adherence emerges as a pivotal, modifiable behavior and a cornerstone in the management of CAD [5]. Pharmacological strategies represent the primary global approach for CAD prevention and management, either solely or in conjunction with procedures like angioplasty and coronary artery bypass surgery [6–8]. Despite the established efficacy of these medications in clinical trials for preventing and managing cardiovascular diseases (CVDs), their practical impact in real-world settings is somewhat diminished, attributed in part to suboptimal medication adherence. Research findings suggest medication non-adherence rates in heart diseases ranging from 33% to over 55% [9, 10]. Notably, a comprehensive meta-analysis involving nearly 2 million patients revealed that only 60% adhered to their cardiovascular medications. Furthermore, poor adherence correlated with a 20% higher risk of cardiovascular events and a 35% elevated mortality risk compared to individuals exhibiting good adherence [11]. The impact of treatment adherence on effectively managing diseases and mitigating adverse outcomes is well-documented [12, 13]. Medication adherence (MA), in theory, refers to the extent to which an individual’s medication-taking behavior aligns with the prescribed regimen by their physician [14]. Non-adherence to medication regimens stands as one of the primary reasons for uncontrolled CAD, resulting in heightened hospitalizations, escalated treatment costs, and a decline in Health-Related Quality of Life (HRQoL) [13, 15, 16]. Studies have indicated a positive correlation between medication adherence and a patient’s acceptance of their illness [17].

Adherence, a multifaceted and intricate concept, is shaped by various factors. Among these, illness perception stands out as a predictive element influencing patient behaviors in managing their condition, particularly concerning medication adherence. Illness perception encompasses the organized beliefs a patient holds regarding their disease, exerting a profound influence on their adaptive responses, health-related decisions, and adherence to lifestyle modifications [18–20]. Acceptance of the illness also holds significance and enables patients to function effectively despite the challenges posed by a chronic disease [2]. Acceptance of the illness involves adapting to living with the illness without judgment, avoidance, or denial, while maintaining engagement in daily activities [17]. It entails active participation in treatment and recovery through medication adherence, prescribed diet, and lifestyle changes [17, 21]. The existing body of research has established connections between medication adherence and the acceptance of illness through various studies. However, comprehensive investigations simultaneously exploring the interplay among acceptance of illness, medication adherence, and illness perception are lacking. Consequently, this study endeavors to assess the degrees of treatment adherence, acceptance of illness, and illness perception among patients diagnosed with coronary artery disease (CAD) seeking treatment at Rouhani Hospitals in Babol, Iran. The primary objective is to elucidate the relationships among these factors within this specific patient population.

## Methods

### Study population and site

This cross-sectional and correlational study spanned from March to September 2023 and focused on individuals diagnosed with coronary artery disease (CAD). The research took place at the Rouhani Hospital’s heart clinic in Babol City, Iran, serving as the designated research site. Notably, the specialized clinics operated daily during morning shifts, excluding holidays. A total 280 individuals diagnosed with confirmed coronary artery disease (CAD) were selected via a convenience sampling method that adhered to predefined inclusion criteria.

### Inclusion and exclusion criteria

The study’s inclusion criteria comprised individuals aged 18 and above, diagnosed with chronic coronary disease displaying at least one vessel involvement of more than 50% as confirmed by angiography or CT angiography, under specialized cardiac care for a minimum of 6 months, and providing informed consent for participation.

Individuals were excluded for participation in the study if they met any of the following criteria: (1) exhibited cognitive impairment, (2) sought facility admission due to an acute episode of diseases that could impede their active involvement, or (3) explicitly declined to participate in the research endeavor.

### Sample size

Considering a confidence level of 95% and a test power of 90%, the determination of the sample size was predicated on an estimated correlation coefficient of 0.2, indicating a significant relationship between the variables. The formula utilized for calculating the required sample size, denoted as ‘n,’ incorporates the confidence coefficient for 95% (z1 = 1.96), the power coefficient for 90% (z2 = 1.28), and the correlation coefficient ‘r.’ The derived equation, n=((z1 + z2)^2 (1 -r^2))/r^2 + 3, factored in an allowance for potential dropout from the study. As a result, the determined sample size for this investigation involving patients with coronary artery disease (CAD) was 280.

### Research instruments

The questionnaire employed in this study comprised four distinct sections: (1) inquiries pertaining to individual, social, and medical factors, (2) assessment of medication adherence, (3) evaluation of illness acceptance, and (4) utilization of the Brief Illness Perception Questionnaire (IPQ-B). A concise overview of the items encompassed within each section is delineated below for reference.

### Sociodemographic and medical factors

The section encompassing individual, social, and medical factors comprised inquiries concerning various participant demographics, including age, gender, income, Job, level of educational, economic Status, marital status, place of residence, BMI (Body Mass Index), intention to stop using drugs and complications.

### Medication adherence

The assessment of self-reported medication adherence employed the eight-item Morisky Medication Adherence Scale (MMAS-8). This tool is known for its simplicity in administration, reliability, and cost-effectiveness in clinical settings. Total scores on the MMAS-8 range from 0 to 8, with scores of 8 reflecting high adherence, 7 or 6 reflecting medium adherence, and < 6 reflecting low adherence [22]. The Persian version of the MMAS has undergone validation in Iran and is extensively utilized by researchers investigating chronic conditions like hypertension and diabetes [23, 24]. To ascertain the tool’s reliability, Cronbach’s alpha coefficient was computed, yielding a value of 0.7 for the MMAS-8, affirming its consistency and reliability as a structured self-report assessment tool for medication adherence.

### Acceptance of Illness Scale (AIS)

We adapted the Acceptance of Illness Scale (AIS) questionnaire of Juczyński [25]. This scale comprises eight statements delineating the adverse implications of poor health, encompassing assessments of limitations imposed by the illness, diminished self-sufficiency, feelings of dependence on others, and reduced self-esteem. Participants rate their present condition on a five-grade scale, ranging from 1 “strongly agree” to 5 “strongly disagree” for each statement. Strong agreement (grade 1) signifies a challenging adjustment to illness, while strong disagreement (grade 5) indicates a higher degree of acceptance of the illness. The total score, ranging from 8 to 40, serves as a comprehensive measure of illness acceptance. Scores below 20 are indicative of low acceptance and adjustment to illness, often correlated with substantial emotional challenges linked to the illness. Scores between 20 and 30 suggest a moderate level of acceptance, while scores above 30 indicate a higher or full acceptance of the illness. The Acceptance of Illness Scale (AIS) has undergone translation into Persian, exhibiting satisfactory psychometric properties [26, 27]. To ascertain item validity, a panel of 15 academic professors evaluated each question using the Content Validity Index (CVI) and Content Validity Ratio (CVR). The CVR values exceeded the threshold determined by Lawashe for each question, indicating acceptable necessity validity across all items. Moreover, the CVI scores, reflecting the simplicity, clarity, and relevance of the questions, surpassed 0.90, affirming their validity from this perspective. Reliability assessment employed Cronbach’s alpha coefficient, yielding an internal consistency of 0.87 for the entire instrument, indicative of robust reliability.

#### Brief illness perception questionnaire (IPQB)

The Illness Perception Questionnaire-Brief (IPQ-B) is a nine-item scale that evaluates an individual’s emotional and cognitive perspectives regarding their illness [28]. Each item, rated on a scale from 0 (minimum) to 10 (maximum), addresses different dimensions of illness understanding. For instance, items 1–5 focus on cognitive aspects related to comprehending the illness, its causes, treatment impact, while items 6-to-8-gauge emotional dimensions, including mood, fear, anxiety, or anger. Item 9 solicits the patient’s opinion on the illness’s cause. The total illness perception score is derived by combining the scores from all items after adjusting scores for items 3, 4, and 8. This total score ranges from 0 to 80, with higher scores (56–80) indicating a more threatening perspective toward the illness, while lower scores (0–27) denote a more optimistic viewpoint and score between 28 and 55 represent moderate level of illness perception [28]. The Farsi version’s validation and localization were conducted by Bazzazian and Besharat, verifying its reliability and validity [29]. Ten medicine faculty members at Babol University of Medical Science confirmed its validity, while Cronbach’s alpha coefficient, measuring internal consistency, demonstrated a high reliability level of 0.82.

Preceding the main study, a pilot test involving 15 randomly selected CAD patients assessed the questionnaire’s content, structure, and clinical relevance. Feedback from this pilot test facilitated minor adjustments to address potential issues, such as confusing language, ensuring the questionnaire’s clarity and relevance.

### Data collection procedure

The study encompassed the entirety of CAD patients available at the clinic during the data collection period. Qualified patients were guided to a designated quiet area. Subsequently, a team of researchers responsible for conducting the interviews provided pertinent explanations and outlined the research’s objectives to the patients and the same team of researchers was involved in data collection procedures. Each question within the questionnaire was verbally presented by the interviewer to all patients, and the interviewer’s colleague marked the corresponding responses in the appropriate columns. On average, completion of each questionnaire required approximately 20 to 25 min.

### Ethical considerations

The study adhered to the ethical principles outlined in the revised Declaration of Helsinki. Approval for the research was obtained from the Institutional Research Ethics Committee of Babol University of Medical Sciences, Babol, Iran (ethical code: IR.MUBABOL.HRI.REC.1401.282). Prior to participation, written informed consent was acquired from all enrolled individuals. Each participant received comprehensive information regarding the study’s objectives and procedures, along with assurances regarding the preservation of their anonymity and confidentiality of their data. Furthermore, participants were explicitly informed of their voluntary participation and their right to withdraw from the study at any stage without any repercussions. A written inform consent has been taken from each participant.

### Statistical analysis

The data collected underwent analysis employing IBM SPSS Statistics version 25.0 (IBM Corp., Armonk, NY: IBM Corp.). Descriptive statistics were employed to delineate the sociodemographic characteristics of the participants. Pearson correlation was utilized to assess correlations between variables, focusing on identifying statistically significant factors. Subsequently, significant factors were integrated into a multivariate linear regression model to account for potential confounding variables. Assessment for normality was conducted through the Kolmogorov-Smirnov test, while collinearity among variables was examined using the variation inflation factor (VIF). A threshold of *p* < 0.05 was set to establish statistical significance.

## Results

All participants enrolled in the study successfully completed the questionnaire, yielding a 100% response rate. Consequently, the analysis included a cohort of 280 eligible patients diagnosed with confirmed coronary artery disease (CAD). The demographic and clinical profiles of these participants are summarized in Table 1. The mean age of the participants was 58.99 ± 10.5 years. Gender distribution indicated that slightly over half of the participants were women (50.4%), and the majority were married (75%), with 36.4% identifying as housewives. Additionally, 84.3% resided with their families, while 8.9% held a university degree. Economic status analysis revealed that 59.3% of participants had an average economic standing. Geographically, most participants lived in urban areas (65.0%). Medication-wise, 74.6% were on oral medications, and 72.1% did not exhibit any complications related to their condition.

**Table 1:** Sociodemographic and Clinical Characteristics of the Participants (*n* = 280)

Variable	Category	N (%)
Age	24–44 Year	22(7.9)
	45–54 Year	70(25)
	55–64 Year	103(36.8)
	65–74 Year	69(24.6)
	>74 Year	16(5.7)
Gender	Female	141(50.4)
	Male	139(49.6)
Level of Education	Illiterate	110(39.3)
	Under diploma	109(38.9)
	Diploma	36(12.9)
	University	25(8.9)
BMI	18.5–25	67(23.9)
	25–30	134(47.9)
	>30	79(28.2)
Economic Status	Weak	81(28.9)
	Moderate	166(59.3)
	Good	33(11.8)
Place of Residence	Urban	182(65)
	Rural	98(35)
Type of drug	Oral	209(74.6)
	Injection & Oral	71(25.4)
Complications	Yes	78(27.9)
	No	202(72.1)
Intention to stop using drugs	Yes	44(15.7)
	No	236(84.3)
Marital Status	Single	15(5.4)
	Married	210(75)
	Divorced	24(8.6)
	Widowed	31(11.1)
Job	Employee (Regular salaried)	25(8.9)
	Worker (Daily wages)	37(13.2)
	Farmer	35(12.5)
	House	102(36.4)
	Self Employed	52(18.6)
	Retired	29(10.4)

Table 2 presents the research outcomes concerning medication adherence, illness perception, and acceptance of illness within the participant pool. The analysis revealed that 61.8% of participant’s MA, demonstrating a mean value of 6.16 ± 1.46. In terms of illness perception, the calculated mean ± standard deviation was 51.82 ± 7.58, indicating a moderate perception of illness within the cohort. Moreover, the mean ± SD for illness acceptance was determined to be 16.98 ± 4.75, signifying a relatively low level of acceptance of the illness among the participants.

To examine relationships between variables, Spearman’s correlation coefficient was employed (Table 2). This analysis indicated a statistically significant positive relationship between acceptance of illness and medication adherence (*r* = 0.435, *p*-value < 0.01).

**Table 2:** The Mean (SD) Scores of Medication Adherence, Illness Perception, and Acceptance of Illness along with correlation between them (*n* = 280)

Variable	Medication Adherence			Illness Perception			Acceptance of Illness		
Possible Scores	0–8			8–80			8–40		
Observed Scores	0–8			28–80			8–27		
Mean ± SD	6.16 ± 1.46			51.82 ± 7.58			16.98 ± 4.75		
Category	Low (< 6)	Medium (6–7)	High (8)	Low (0–27)	Moderate (28–55)	High (56–80)	Low (< 20)	Moderate (20–30)	High (> 30)
N (%)	109 (38.9)	100 (35.7)	71 (25.4)	---	204 (72.9)	76 (27.1)	209 (74.6)	71 (25.4)	---
Correlations among outcome variables									
Medication Adherence	1			-0.022			0.435**		
Illness Perception	-0.022			1			0.107		
Acceptance of Illness	0.435**			0.107			1		

** significance at 0.05 level; % - percentage; SD – standard deviation

The findings from the logistic regression analysis aimed to explore factors associated with medication adherence (MA). The analysis revealed that participants without formal education exhibited a medication non-adherence rate 3.81 times that the participants with a university education, also displaying statistical significance (*p* = 0.01). Furthermore, participants who were on oral medication and divorced were 1.73 and 4.05 times more adherence to the treatment than the participants who were on injectable and widowed respectively (*p* < 0.05). However, it was observed that other variables examined in the analysis did not show a significant relationship with medication adherence (Table 3).

It is noteworthy that the logistic regression analysis did not find any significant relationship between illness perception and variables examined (Table 3).

The regression analysis uncovers notable associations influencing the acceptance of illness among CAD patients. Gender emerges as a significant factor, with women exhibiting a disease understanding score 0.03 units lower than men, marking a statistically significant difference (*p* = 0.03). Education levels also play a substantial role, with single and divorced patients demonstrating significantly lower acceptance of illness (OR = 0.068 and 0.148, respectively). Additionally, patients with an intention to discontinue medication exhibited a likelihood 0.28 times lower than those without such intentions to adhere to their drug regimen (*p* < 0.05) (Table 3).

## Discussion

The primary objective of this investigation was to explore the factors influencing medication adherence among patients diagnosed with coronary artery disease (CAD), with a particular focus on the relationship among medical adherence, illness perception and acceptance of illness. CAD represents a significant healthcare challenge globally and its prevalence escalates with age, impacting a considerable portion of the population [30]. Effective management of CAD critically relies on adherence to prescribed medications [31]. Non-adherence to medication can result in adverse clinical consequences, notably heightened mortality rates and increased frequency of hospital admissions within this patient cohort [32]. Medication adherence in CAD patients is a complex phenomenon shaped by numerous determinants. Identifying these factors is crucial, given that non-adherence significantly compromises treatment effectiveness and escalates healthcare costs associated with chronic conditions demanding long-term therapeutic interventions [33]. This study stands as the pioneering endeavor to scrutinize the nexus between illness perception, acceptance of illness, and medication adherence within CAD patients.

**Table 3:** Factors associated with medicine adherence, illness perception and acceptance of illness

Variable	Medicine adherence				Illness perception				Acceptance of illness			
	Est. B	OR	*p*-value	95% CI	Est. B	OR	*p*-value	95% CI	Est. B	OR	*p*-value	95% CI
Age	-0.004	0.99	0.7	0.95–1.008	0.015	1.01	0.28	0.98–1.04	-0.03	0.97	0.06	0.94–1.002
BMI	0.005	1.005	0.83	0.97–1.019	0.017	1.02	0.57	0.95–1.08	0.023	1.02	0.5	0.95–1.09
Level of Education (Illiterate)	1.145	3.14	**0.02**	1.158–8.53	0.01	1.01	0.98	0.31–3.25	-3.11	0.04	**0.002**	0.006–0.318
Level of Education (Under diploma)	0.534	1.7	0.3	0.61–4.71	0.37	1.44	0.54	0.43–4.83	-2.68	0.06	**0.007**	0.01–0.48
Level of Education (Diploma)	0.56	1.75	0.31	0.59–5.19	0.441	1.55	0.52	0.4–5.98	-1.12	0.325	0.3	0.03–2.72
Gender (Female)	-0.408	0.66	0.12	0.39–1.119	0.167	1.18	0.61	0.62–2.24	-0.77	0.46	**0.03**	0.22–0.95
Economic Status (Weak)	0.413	1.51	0.33	0.65–3.5	0.103	1.1	0.84	0.39–3.15	-0.1	0.9	0.86	0.27–2.99
Economic Status (Moderate)	0.547	1.72	0.16	0.79–3.75	-0.095	0.9	0.85	0.34–2.36	-0.214	0.8	0.7	0.26–2.45
Place of Residence (Urban)	0.03	1.03	0.9	0.63–1.67	0.504	1.65	0.08	0.93–2.93	0.075	1.07	0.822	0.56–2.06
Type of drug (Oral)	0.549	1.73	**0.04**	1.01–2.96	-0.405	0.66	0.24	0.33–1.31	-0.392	0.67	0.29	0.32–1.39
Complications (Yes)	-0.482	0.61	0.09	0.35–1.08	0.103	1.1	0.76	0.55–2.2	0.66	1.94	0.122	0.83–4.53
Intention to stop using drugs (Yes)	0.201	1.22	0.55	0.624–2.39	-0.65	0.52	0.1	0.23–1.15	-1.25	0.28	**0.006**	0.11–0.7
Marital Status (Single)	0.198	1.21	0.75	0.35–4.22	0.241	1.27	0.75	0.28–5.62	-2.69	0.068	**0.002**	0.01–0.37
Marital Status (Married)	0.194	1.21	0.6	0.58–2.51	0.404	1.49	0.35	0.64–3.5	-0.102	0.9	0.83	0.33–2.4
Marital Status (Divorced)	1.4	4.05	**0.01**	1.34–12.22	0.484	1.62	0.48	0.42–6.25	-1.91	0.148	**0.01**	0.03–0.64
Job (Employee)	-0.021	0.98	0.97	0.32–2.99	0.258	1.29	0.72	0.3–5.41	1.32	3.77	0.1	0.74–19.23
Job (Worker)	0.345	1.41	0.49	0.52–3.78	-0.438	0.64	0.46	0.2–2.08	0.067	1.06	0.91	0.31–3.63
Job (Farmer)	-0.658	0.51	0.19	0.19–1.4	-0.095	0.91	0.87	0.27–2.97	1.18	3.27	0.077	0.87–12.17
Job (House)	-0.158	0.85	0.72	0.35–2.08	-0.155	0.85	0.77	0.29–2.53	1.17	3.23	0.056	0.97–10.75
Job (Self Employed)	-0.106	0.89	0.82	0.35–2.25	0.207	1.23	0.72	0.38–3.93	0.37	1.45	0.56	0.41–5.12

*P < 0.05, Est.: Estimated, Level of Education: University as baseline. Gender: Male as baseline. Economic Status: Good as baseline. Place of Residence: Rural as baseline. Drug type: Injection as baseline. Marital Status: Widowed as baseline. Job: Retired as baseline

The study’s findings revealed that a majority of participants (61.8%) adhered to their prescribed medication, aligning with prior research outcomes [34–36]. However, adherence rates display substantial variability across studies. For instance, adherence rates among CAD patients fluctuated between 42.9% and 70.9%, with reported non-compliance rates soaring to 70% in certain studies [10, 37]. Furthermore, following cardiac angiography, only 32% of patients exhibited high adherence to medication regimens [38]. In a study encompassing patients with chronic diseases such as diabetes, hypertension, or both, in Saudi Arabia, a medication adherence rate of 76.44% was observed [39]. Typically, medication adherence within populations experiencing CAD patients averages around 43% [40]. However, to attain favorable treatment outcomes in this specific group, maintaining adherence levels at 80% or higher is advocated [41]. It is imperative to account for variations in demographic, clinical variables, and the diverse methodologies employed in questionnaire assessments across previous studies, as these factors may contribute to the disparate adherence outcomes observed.

Gender emerged as a significant non-modifiable determinant associated with self-care adherence in this investigation. Notably, females demonstrated a 0.48 times lower likelihood of exhibiting good adherence compared to their male counterparts within this study. Similar patterns were evident in parallel research conducted in South Africa and the Netherlands, wherein men exhibited higher tendencies toward treatment adherence compared to women [42, 43]. One potential explanation could be that women tend to encounter a higher frequency of adverse reactions to medication compared to men [39]. Therefore, it is important for healthcare providers to consider these factors when prescribing medications to women and to tailor treatment plans accordingly to minimize the risk of adverse reactions. However, findings by Ok et al. contradicted this trend, reporting that women displayed better adherence to treatments than men [44]. Intriguingly, several studies presented conflicting outcomes regarding gender disparities and their association with treatment adherence [45–47]. Specifically concerning individuals with heart failure (HF) and other cardiac conditions, a distinct trend emerged indicating that women are more susceptible to experiencing psychosocial distress and have a greater need for social support compared to men [48].

This dynamic, characterized by higher psychological distress and reduced social support, was noted in several studies as being correlated with suboptimal self-care practices [49, 50]. Hence, while gender plays a discernible role in medication adherence, the influence of psychological distress and limited social support appears to be pivotal factors, particularly among women with cardiac conditions.

The current investigation has revealed a moderate level of illness perception, demonstrating a negative correlation with medication adherence. Specifically, individuals perceiving their illness as a severe and menacing condition exhibited decreased adherence to prescribed medications. This aligns with Saarti et al.‘s study on hypertensive patients, where those with poorer medication adherence displayed higher average illness perception scores, despite a lack of a direct relationship between illness perception and adherence in their findings [51]. Correspondingly, Gauro et al.‘s study in Nepal unveiled an inverse association between illness perception and cardiovascular health behavior [52]. highlighting the potential for psychological interventions targeting perceptions of illness to bolster disease management practices and foster adherence to health-promoting behaviors [53]. Conversely, Doust Mohammadi et al.‘s research underscored illness perception as a pivotal and influential factor in medication adherence among patients with chronic illnesses [19]. They advocated for strategies aimed at fortifying and augmenting patients’ illness perception as fundamental components within educational interventions geared toward enhancing medication adherence. Consequently, in light of these collective study findings, the imperative emerges for the development of comprehensive training initiatives and health assessment programs. These programs should not only impart knowledge and lifestyle modifications but also concentrate on elevating patients’ comprehension of their illness and its associated risk factors in the context of coronary artery disease (CAD) [54]. The outcomes of our study echoed the findings by Monika Obigelo, indicating that a majority of chronic heart failure patients exhibited a low Acceptance of Illness Scale (AIS) score, attributable to the persistent nature of the condition [55]. Conversely, Agnieszka Pluta’s investigation presented contrasting results to ours, revealing that 62% of their study participants displayed a high AIS [56]. Similarly, other studies reported varied AIS percentages ranging from 45 to 62.0%, averaging at a moderate level [57, 58]. Certainly, the observed disparities could arise from the utilization of distinct assessment tools, variations in the demographics such as age groups studied, and differences in the settings of the respective research endeavors. However, it’s unequivocal that acceptance of illness stands as a pivotal determinant influencing the prognosis within the realm of coronary artery disease (CAD).

While no specific study exploring the nexus between illness acceptance and medication adherence was retrievable, Turen et al. uncovered a statistically significant and positive correlation between levels of illness acceptance and medication adherence [59]. Remarkably, our study corroborated these findings, delineating a noteworthy positive correlation between acceptance of illness and medication adherence among individuals diagnosed with coronary artery disease (CAD). These findings hold promise in shaping individualized care strategies for diverse patient cohorts. Access to comprehensive insights regarding self-perceived health status and its acceptance could significantly enhance the quality of care extended to patients, particularly those grappling with chronic ailments. Tailoring interventions based on these nuanced understandings may effectively optimize patient outcomes and their overall care experiences.

### Limitation

The present investigation is circumscribed by various constraints. The primary reliance on patient self-reporting specially, participant’s economic status and complications during interviews stands as the cornerstone of data acquisition. Consequently, the inherent limitations of recall bias and the potential for patients to inadvertently overlook significant information may exert an influential effect on the overall study findings. Moreover, the absence of multicenter data aggregation, poses challenges in extrapolating the study’s conclusions to the broader populace. Additionally, the study’s inherent nature as a cross-sectional analysis, devoid of subsequent follow-up evaluations, engenders limitations in comprehensively assessing the precise level of patient adherence to medication. This design choice also complicates the ability to establish a definitive causal relationship between variables under scrutiny.

### Implications

Given the significance of managing chronic illnesses, where medication adherence stands as a pivotal factor, assessing and enhancing patients’ illness perception levels, alongside improving their capabilities, holds promise in bolstering medication adherence. Health professionals, particularly physician, nurses and pharmacists, with their frequent patient interactions and active involvement, possess a significant potential to impact this realm. Their role involves evaluating patients’ illness perception and capacities, thereby contributing significantly to augmenting medication adherence. Moreover, our study findings underscore the influence of certain demographic and clinical variables on these pivotal factors. Specifically, lower educational attainment and advancing age emerged as factors influencing medication adherence. Consequently, nurses should allocate focused attention and tailored training to this demographic within their care, addressing these pertinent issues accordingly.

## Conclusion

The current study demonstrated the impact of behavioral factors, specifically illness perception and acceptance of illness, on medication adherence among CAD patients. The participants exhibited moderate levels of illness perception and low to moderate acceptance of their illness. Additionally, only a quarter of the participants adhered to the prescribed drug regimen. The study highlights the imperative to enhance medication adherence, foster acceptance of illness, and positively influence illness perception among coronary artery disease (CAD) patients.

Recognizing that effective chronic disease management should not solely rely on patient effort, health professionals—particularly nurses and pharmacists—hold a pivotal role in this endeavor. Nurses possess the capacity to assess patients’ illness perception, anticipate their acceptance to illness, and take actionable steps to bolster it. While, pharmacists can enhance medication adherence among CAD patients through education, counseling, medication management, monitoring, collaboration with the healthcare team, and behavioral interventions. Nurses and pharmacists multifaceted involvement and interventions can substantially contribute to improved outcomes in the management of chronic conditions like CAD.

## Data Availability

Data associated with this publication can be shared with a reasonable request to the corresponding author.
